# Making the APPropriate choice: Utilization of a smartphone application to optimize antimicrobial decisions among internal medicine trainees

**DOI:** 10.1017/ash.2022.345

**Published:** 2022-12-09

**Authors:** Thomas R. Brooke, Herman O. Pfaeffle, Walter O. Guillory, Sorana Raiciulescu, Roseanne A. Ressner

**Affiliations:** 1 Internal Medicine, Walter Reed National Military Medical Center, Bethesda, Maryland; 2 Biostatistics Consulting Center, Uniformed Services University, Bethesda, Maryland; 3 Infectious Diseases, Walter Reed National Military Medical Center, Bethesda, Maryland; 4 Department of Medicine, Uniformed Services University, Bethesda, Maryland

## Abstract

Utilization of a smart phone application paired with a time-spaced learning curriculum was investigated to determine its impact on antimicrobial stewardship practice among internal medicine trainees. Stewardship behaviors increased, barriers decreased, and trainees had increased confidence in managing common infectious disease syndromes after the intervention.

Antimicrobial stewardship (AS) remains a high priority for healthcare institutions and government regulators. Widespread availability of smartphone applications (app) makes it logical to leverage technology to improve stewardship. Technology-based interventions have primarily focused on clinical decision support systems within the electronic medical record.^1^ However, studies have demonstrated apps are helpful, easy to use, and improve guideline adherence.^2^ Publishing antibiograms on an app can promote wider adoption while decreasing both broad-spectrum antimicrobial usage and antimicrobial resistance.^3,4^

Education is a cornerstone of comprehensive stewardship efforts.^5^ Early active rather than passive intervention is key in changing prescribing behavior.^6^ More specifically, time-spaced educational interventions have shown improved levels of learning and retention.^7^ Training programs utilize apps for teaching, evaluating, and tracking operative experiences have shown improved handoff communication and efficiency.^8^ Not all smartphone app interventions, however, show improved outcomes compared to traditional e-learning platforms.^9^ The efficacy of app-based AS methods to shape antimicrobial prescribing practices is still largely unknown, especially in training programs. We sought to determine the impact of combining an AS smartphone app with a time-spaced learning curriculum within an internal medicine (IM) training program.

## Methods

**Fig. 1.**
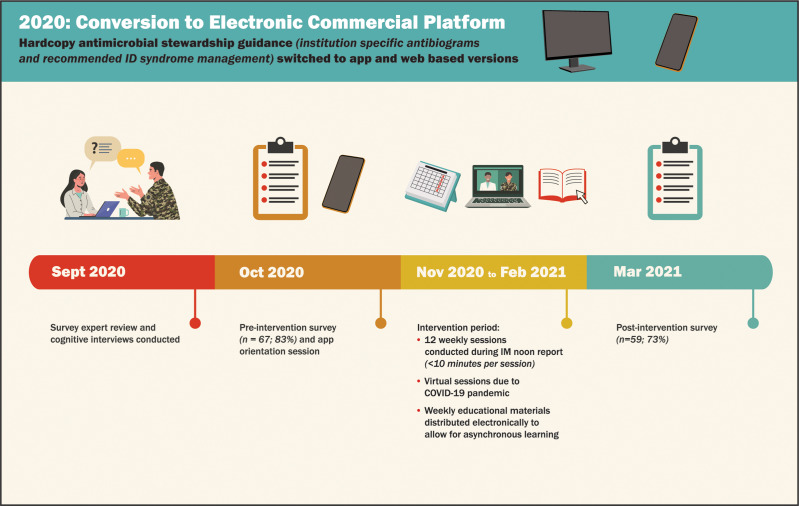
Phases of project implementation.

A prospective, observational, quality-improvement study was conducted at Walter Reed National Military Medical Center (WRNMMC) among IM and dual IM-psychiatry trainees. Each participant was assigned a random unique identifier code (UIC). Study personnel were blinded to the codes, which anonymously linked pre- and postintervention survey responses. External survey reviews and iterative cognitive interviews were utilized.^10^ Figure 1 depicts the stages of project implementation. Education sessions were centered on AS concepts in relation to managing a variety of infectious disease (ID) syndromes (Table 1) while reinforcing app usage as a clinical decision tool. The education sessions implemented active learning techniques to maximize participation.

**Table 1.** Participant Identified Clinical Domains Where AS App Content Directly Improved Clinical Management Abilities^a^

Clinical Domain	%
Know local resistance patterns via an antibiogram	67.8
Manage UTI in women	61.0
Switch from IV to PO antimicrobial therapy	61.0
Manage UTI in men	57.6
De-escalate therapy (ie, go from broad to more narrow or directed therapy)	55.9
Manage community-acquired pneumonia	50.8
Manage skin and soft-tissue infections	49.2
Manage hospital- and/or ventilator-associated pneumonia	47.5
Manage catheter-associated UTI	45.8
Manage intra-abdominal infections	44.1
Appropriately order weight-based vancomycin	42.4
Manage *S. aureus* bacteremia	42.4
Manage *C. difficile*	40.7
Manage febrile neutropenia	35.6
Manage CNS infections (ie, bacterial meningitis and encephalitis)	30.5
Manage asymptomatic bacteriuria	25.4
Initiate an evaluation of a penicillin or β-lactam allergy for potential delabeling	23.7
Manage acute rhinosinusitis	22.0

Note. UTI, urinary tract infection; PO, oral; IV, intravenous; CNS, central nervous system

a Participants could choose >1.

Anonymous survey data were analyzed using SPSS version 27 software (IBM, Armonk, NY). Paired *t* tests were used to compared pre- and postintervention responses. For comparisons between interns and residents, 2-sample *t* tests, with or without the Welch correction where appropriate, were used. The participant UIC ensured that no duplicative responses were included.

## Results

Among 81 eligible trainees, 59 (73%) completed both the pre- and postintervention surveys and were included in the analysis. Responses were comprised of 23 postgraduate year (PGY) 1 participants (39%), 18 PGY2 participants (31%), and 16 PGY3 participants (27%). Also, 1 participant was a PGY4 and 1 participant did not identify a training level. Self-reported app usage increased from 42% to 90%. Moreover, 95% felt that the the app was better than the preceding hard-copy version, and 88% felt that it was easy to use. Using a Likert scale, trainees self-assessed their use of and familiarity with antimicrobial stewardship concepts. Before and after the intervention, trainees had a significant (*P* < .05) increase in their familiarity with the antibiogram, accounting for the antibiogram in clinical decision making, referencing the app, and utilization of an antimicrobial timeout at 48 hours. More specifically, 68% felt that the app directly improved their knowledge of local resistance patterns via the antibiogram. Antibiogram use when making antimicrobial decisions increased from 34% to 90%. Trainees felt that the app directly improved their ability to manage a variety of ID syndromes (Table 1), and confidence in management significantly improved between the pre- and postintervention surveys (*P* < .001) across all ID syndrome categories. Specifically, interns indicated a higher increase in confidence than residents for febrile neutropenia, hospital- and ventilator-acquired pneumonia, and familiarity with the antibiogram. Lack of knowledge (58%), time required to make an informed decision (57%), deference to seniority (49%), and established habits and/or cultural practice (39%) were the most commonly self-identified initial barriers to stewardship (participants could select more than 1). After the intervention, lack of knowledge and time required to make an informed decision decreased statistically significantly to 44% (*P* < .05) and 41% (*P* < .01), respectively. Only 5% identified patient and family pressure as a barrier to stewardship. If the app was not utilized at the point of clinical decision making, forgetting to use the app (42%) and time (13%) were the most frequently identified barriers. When evaluating the impact of weekly education sessions, 75% felt that the sessions had at least a moderate impact on fortifying their AS knowledge, and 20% reported high or extremely high impact.

## Discussion

The Infectious Diseases Society of America (IDSA) and Joint Commission endorse clinician education in AS, but AS programs are often overtasked and underresourced. Most providers own a smartphone device; thus, app use is an accessible and attractive tool for sustainable AS initiatives. Within an IM residency program, an AS app reinforced by an educational curriculum effectively improved AS fundamentals and confidence in clinical ID management while decreasing barriers. Moreover, in just 12 weeks, lack of knowledge and time to make an informed decision were barriers that decreased significantly. Addressing knowledge gaps can empower trainees to tackle deference to seniority and break established habits and cultural practice. PGY1 participants demonstrated the greatest changes in several categories. Larger knowledge and experience gaps are expected in the PGY1 year, which supports the implementation of AS education strategies as early as possible. Embedding minimal time at the beginning of IM academics makes the curriculum highly compact, feasible, flexible, and reproducible over time.

Our study had several limitations. Although all WRNMMC providers have app access, the impact was only evaluated in one training program at a single institution. The impact across other training programs or institutions is a point for further investigation. Second, the COVID-19 pandemic necessitated virtual education sessions. Face-to-face sessions generally foster more active participation, so it remains unclear how this affected the study. Similarly, the intervention was performed in the middle of the academic year, so it is unclear whether its effectiveness would have been greater if it had been implemented at the start of the academic year. Third, only self-perceived improvement in practice was identified. Qualitative outcomes may lead to overestimating the intervention impact because participants may seek to endorse an improvement in stewardship and clinical performance over time. Due to mixed non-IM providers on ward teams and trainee rotation to outside institutions, prescribing practice outcomes and the sustainability of the findings in our study were not evaluated. Lastly, informatics data, such as page views, most visited content, and average session duration, to assess end user needs were only available when users opted in. Our institution’s servers blocked third-party app analytics, so these data were not available. These data might have informed the AS program regarding how to further tailor app information and/or shape curriculum content. Future directions include reproducing the curriculum with face-to-face education sessions at the start of the academic year with the inclusion of additional training programs.

The ready accessibility of a tailored smartphone app is an AS force multiplier where manpower resources are often constrained. The foundations for optimal antimicrobial prescribing practice should be taught early to provide the greatest amount of influence over time. As such, an emphasis should be placed on providing AS tools and education at the earliest point possible in medical training.

In summary, AS app use in conjunction with education reinforcement is an effective way to influence prescribing practices and decrease barriers to AS while bolstering knowledge, incorporation of resistance patterns, and ID syndrome management skills. This project could serve as a blueprint for a multifaceted AS curriculum utilized across a variety of training programs, institutions, and within undergraduate and graduate medical education.
